# On the Functions of the Nervous System

**Published:** 1815-01

**Authors:** C. W. Smerdon

**Affiliations:** Member of the Royal College of Surgeons.


*For the Medical and Physical Journal.*

### *On the Functions of the Nervous System*; by C. W. SMERDON, Member of the Royal College of Surgeons.

TO think and to compare are the operation of faculties which are peculiar to man, and it is to the proper application of these that the human mind is indebted for its highest attainments. To draw conclusions on the nature of causes that are only known to us by their effects, by comparing them with others which are familiar to us, and whose effects are similar, is, I conceive, a very rational method of gaining knowledge: by this mode of theorising, the intricacy of science is considerably lessened; and, where phenomena do not admit of explanation by experimental inductions, we shall, by such means, approximate as near as it is possible to truth. From this source, natural philosophy has received the most considerable of its improvements. By the falling of an apple from a tree, the immortal Newton gained his first ideas of gravity, by which, by the curve which a body forms when thrown into the air, and by centrifugal power, the evolution of the planets around the sun, as their common centre, is very rationally explained. The simple experiment of pouring water on a body placed in a basin, proves the refractibility of the rays of light, and consequently explain the phenomenon of twilight; and, by reasoning on the resemblance which apparently exists between a common culinary fire and the sun, we are indebted for no inconsiderable portion of what we know of this luminary.

In the following observations, some of the functions of the nervous system are attempted to be explained on this principle, nor can such a plan be considered as irrational, since we know that the anatomist, even after the most attentive dissection of the brain, is still left to wonder and to doubt; nor has any clue hitherto been discovered, which can lead to the physiology of that organ—the human intellect, that masterpiece of creative wisdom, is still enveloped in impenetrable mystery.

It is an axiom which I think cannot admit of doubt, that all organic actions depend, first, on the living principle; secondly, on a peculiar structure; and thirdly, on stimuli: and it is equally clear, that actions are powerful in proportion to the quantity of stimuli applied.

All matter animated with life, and possessing the property of contracting on the application of stimuli, seem to be endowed with a certain principal or spring of action, destined by nature to be exquisitely sensible to peculiar impressions: this principal is the irritability of the modern physiologist.

The

The nature or cause of irritability is totally unknown, nor is it my intention to enter into any disquisition on a subject which is not within the reach of the human intellect: it may or it may not be the living principal, but it is evidently inherent in the part which possesses it, and not dependant on structure or organization.

The actions, then, of all organic bodies are derived from the reciprocal action of these two principals, one of which is inherent and the other derivative.

It is also an established fact, that nature has adapted to every contractile part a natural stimulus; thus, for instance, the peculiar stimulus of the voluntary muscles is the nervous energy, while that of the involuntary is either the secretion or the contained matters of viscera to which they are attached, or the blood. As examples of these, I may instance the muscular coats of the stomach, intestines, urinary bladder, &c. and the heart and arteries.

An impulse or thought, whether it springs up of itself, or emanates from external objects, is to the mind what the blood is to the heart and arteries, the urine to the urinary bladder, or the gastric fluid to the stomach; it acts on the organic structure of the brain as its peculiar stimulus, and if motion be the object in view, the nervous energy (the supposed product of this action) is propelled, with the velocity of electricity, along the fibrillæ of the nerves to such muscles as are destined to be put in motion; and, being the natural stimulus of the muscular fibres, it immediately acts on their irritability, and to this action I assign the cause of muscular contraction.

To illustrate this point, let us suppose that the vitality of a muscle could be effectually destroyed, without destroying its organization, or the life of other parts of the animal; is it reasonable to suppose that the will could exercise any controul over this muscle, or that the application of nervous energy or any other stimulant could make it contract? It would be the height of folly or ignorance to suppose so, and still why should not these phenomena take place, seeing that the nerves are still entire, and consequently the influx of nervous energy from the brain to the muscle not impeded? The answer is plain: the presence of another principal besides nervous fluid is essentially necessary to produce the contraction of muscular fibres, and this principal is life or irritability; and hence it must follow, that, if two agents be necessary to produce a certain effect, it must be brought about by their reciprocal action on each other.

That the faculties of the mind, in common with all the organs of the body, have their natural stimuli, is sufficiently demonstrated demonstrated by the well-known effects of some of the passions. These I shall consider as inordinate stimuli, the principal of which are joy, surprise, fear, grief, suspense, anger, &c. To prove by familiar examples that these do act as stimulants, let us suppose that a man, whose mind is in a state of great exhaustion, so that he is irresistibly led to fall asleep, receives the news of an unexpected accumulation to his fortune, or is thrown into imminent danger by the house he inhabits suddenly catching fire; here we have an example of excessive joy on the one hand, and of fear on the other;—in either case sleep will immediately forsake him; his mind will become inordinately acted upon, because each of these stimuli are rendered powerful by the causes from whence they spring; and, the nervous energy consequent on their action being also proportionably powerful, the muscular fibres will receive an extraordinary degree of strength: hence it is that we are enabled to make such powerful efforts to avoid causes that threaten our immediate existence, and to undergo excessive bodily exertions which in ordinary states of our mind would be alone sufficient to destroy life. The stimulus of enthusiasm is peculiar to man, and would seem to be an emanation from the soul, since it generally makes him aspire to deeds of the nobler kind: hence the stimulus of enthusiasm, when moderated by reason, elevates the mind to a higher degree of perfection than it possibly could acquire by any other means; without it, human life would never have towered above mediocrity, and the wonders of nature, which the sciences have disclosed, would always have remained enveloped in their original darkness. Unfortunately, when this stimulus is applied in an inordinate degree, or to a mind originally weak, it seems to occasion an altered action, in consequence of which, wrong ideas either spring up of themselves, or when emanating from a real object, are falsely associated, so that the judgment and the imagination become perverted, and the person is driven from society by the general consent of its members, because he thinks and acts differently to them.

If we judge of the functions of the brain and nervous system, by what we know of the economy of arteries and their secretions, the comparison in some instances would bear us out in supposing that there is a strong analogy between them. We know, for instance, that peculiar structures or glands are necessary for peculiar secretions—the liver, the kidneys, the salivary glands, are all so many secreting apparatusses, and produce fluids materially differing from each other. The nerves destined for the senses all require different structures. The wonderful mechanism of the ear, and the the manner in which the auditory nerve is distributed, differ as much from the eye and the formation of the retina, as sound does from sight; and the extremities of the fingers, and the papillæ of the tongue, constituting the organs of touch and taste, are structures evidently peculiar to each other. Why is it, I will ask, that bile is not secreted in the kidneys, and urine in the liver? Is it because a different sort of blood is sent to each of these organs? This we know is by no means the case; and it is highly probable that nature pursues the same laws in the production of all the secreted fluids. The reason, I think, is obvious—the structure by some unknown power inherent in itself produces the variety, and, as the liver is totally different in this respect from the kidneys, so it transforms decomposed blood into bile, while the latter secern the same sort of fluid into urine. By the same argument we may ask, why is it that we never see with our ears, or hear with our eyes, or smell with our tongue? Is the nervous fluid, as it emanates from the brain, totally different in each of these parts, thus producing different effects? If we may be allowed to judge of all the senses by the nerves that supply the papillæ of the tongue or finger, we might place a negative to this question with as much plausibility as to the same respecting the secretion from arteries. To the first of these organs, two branches of a pair of nerves may be easily traced, which send ramuli to the sublingual gland, while every other branch of these nerves are distributed about the face, teeth, &c. following the course of the internal maxillary arteries. Now, who will say that taste resides in the sublingual gland as well as in the tongue, or in such portions of the face on which the rami of the fifth pair are distributed? and still this evidently would be the case, if the external senses were produced entirely by the matter which emanates from the brain.

The inferences which I would draw from the conclusions are obvious, for, as the blood which is sent to different secreting organs is changed into as many different fluids by powers inherent in the organs themselves, so with respect to the nervous system, as it regards the external senses. The organs fitted for the development of these may make some alteration, or occasion some action on the nervous matter which they receive from the brain, when their peculiar stimuli are applied to them; thus, the stimulus of the retina is light, of the olfactory nerves minute particles arising from odorous bodies, and so on, by which means a confusion of the senses may be prevented, and the nerves of each organ fitted for the reception of such impressions as they are destined to receive. It may be asked, indeed, why do the optic and olfactory olfactory nerves arise from remarkable eminences, if they derive no peculiarity from them? I cannot answer this question, but I will myself ask, what advantage do the nervi pathetici derive from having their apparent origin in the testes of the brain? Are not these eminences equally as remarkable as the thalami or the corpora striata? and still we know that the pathetici are merely nerves of sensation and loco-motion. The sense of touch is as distinct as any of the others, still, as far as we are able to judge, the nervous filaments which supply the papillæ of the fingers, and those which are destined for loco-motion to the arm, are derived from one common origin.

The cause or causes of the temperature of animals, as well as the source of carbon in the blood, are also referable to the nervous system: of them, therefore, I shall consider in your next, and, in doing so, endeavour to point out a new mode of theorising, that will lead to a rational explanation of these important theological subjects.

*Clifton, Bristol;* C. W. SMERDON.
*Nov.* 12, 1814.

